# Terry L. Erwin and the race to document biodiversity (1940–2020)

**DOI:** 10.3897/zookeys.1044.68652

**Published:** 2021-06-16

**Authors:** Grace P. Servat

**Affiliations:** 1 Smithsonian Conservation Biology Institute. Center for Conservation Education & Sustainability (CCES), 1100 Jefferson Dr. 3123, Washington D.C, 20560 – MRC 705, USA Smithsonian Conservation Biology Institute. Center for Conservation Education & Sustainability Washington D.C United States of America

**Keywords:** Carabidae, canopy studies, fogging, species diversity, tropical rainforest

## Abstract

Terry Erwin’s race to document arthropod diversity inspired taxonomists, systematists, ecologists, evolutionary biologists, and the conservation community at large, as his curatorial work of more than 50 years at the Smithsonian’s National Museum of Natural History and prolific publication record attests. The biography compiles public records, publications, as well as personal memoirs to describe the context in which Erwin’s studies with carabid beetles evolved as formalization of concepts, such as biological diversity, megadiverse countries, biodiversity loss, and conservation biology, will become central for science in the upcoming years. Awareness to explore new frontiers such as the forest canopy and Erwin’s studies in tropical forests, his easy-going personality, and dedicated mentoring attracted colleagues, students, and the general public, making him one of the leaders of tropical biology in the world.

## The wonder years

**Figure 1.**
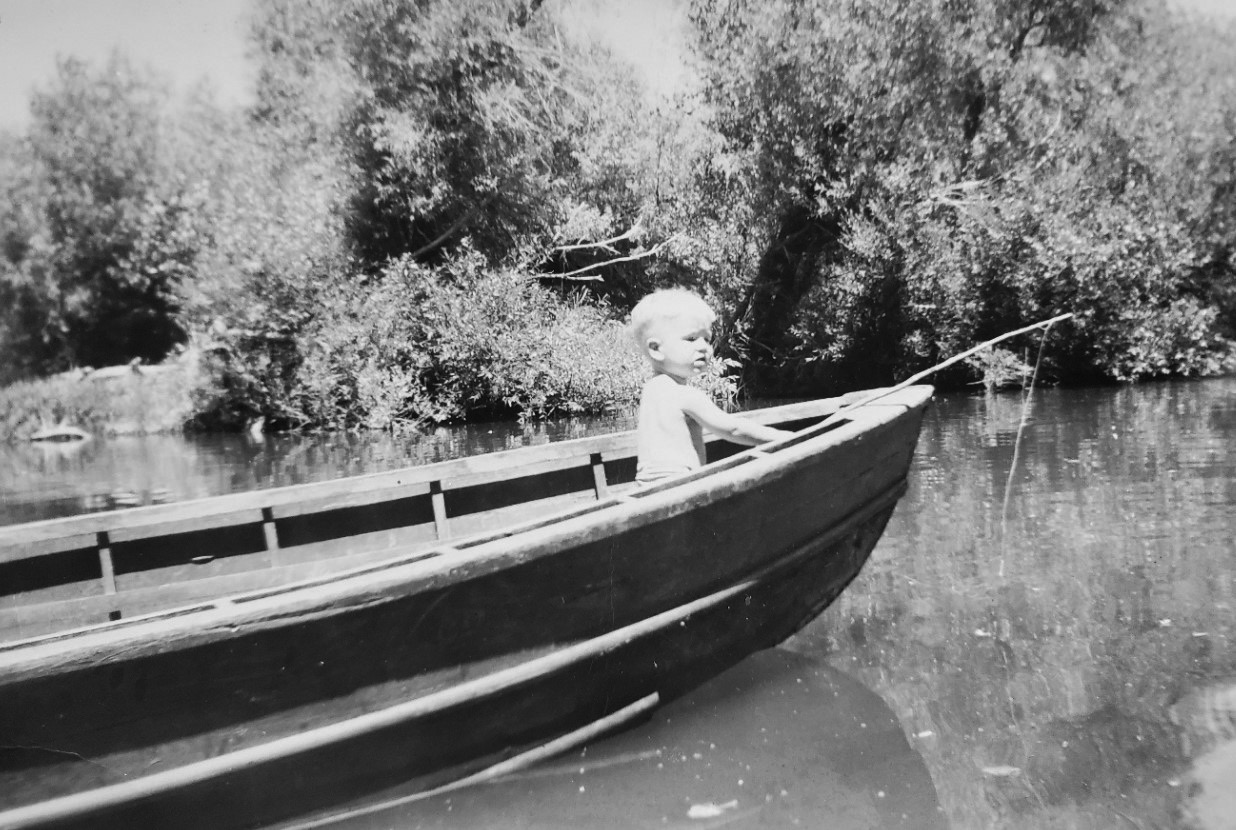
Sense of self and wonder of nature. Terry’s fishing near lake Tahoe and Giant Sequoia forests.

Terry’s early exposure to nature was nurtured by his outdoorsy family and his surroundings. He grew up in the small town of Vallejo, California, located in the region of the San Pablo and San Francisco Bays, the Coastal Redwood forests, the Napa Valley, and the Sierra Nevada. Terry (and sisters Jeani and Toni) spent his childhood summers camping and fishing with his grandfather in the magical world of Giant Sequoia forests, an experience that inspired his sense of self and wonder of nature (Fig. 1).

**Figure 2.**
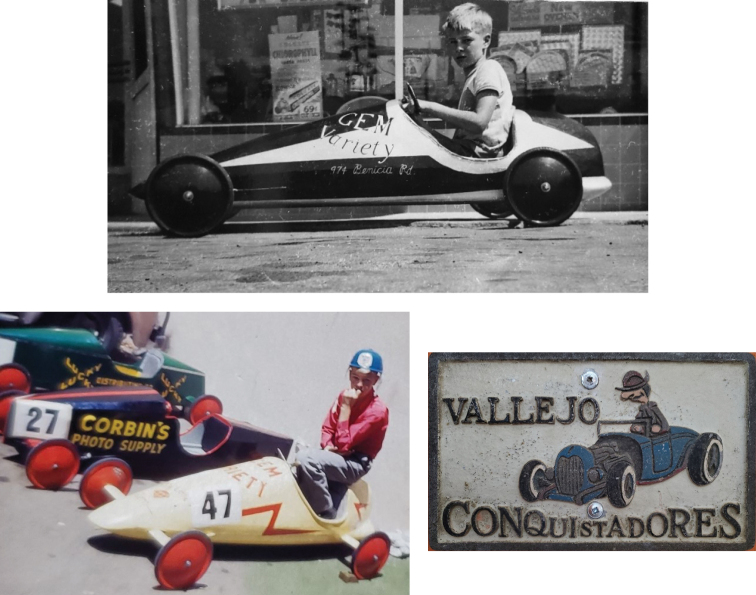
**A** Terry built his own hot rod when he was 12 years old **B** waiting for the race to start **C** car plate of the Conquistadores Club. Terry treasured the plate and keep it in his office throughout his life.

**Figure 3.**
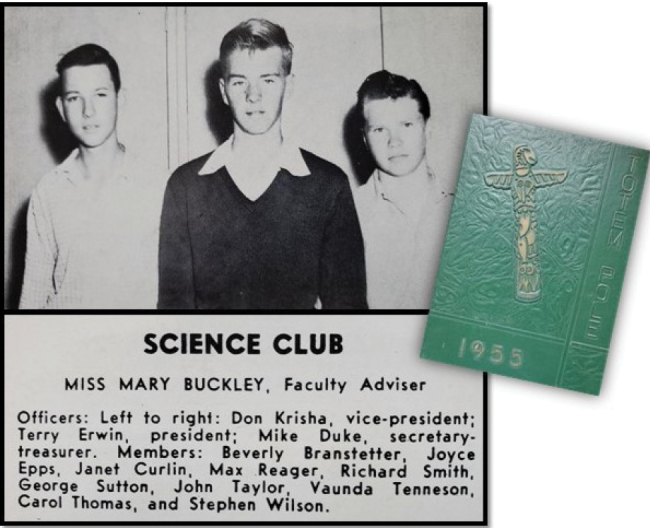
Besides having part time jobs and car racing, waterskiing, and rock and rolling, Terry ‘the overachiever’ also was a good student inclined to science (photograph: Yearbook 1955).

Terry’s mother, June Gephardt, was a government clerk, and his father, Ed Erwin, worked at Mare Island Shipyard building submarines during WWII and the Cold War. Ed was also a racing car driver in the California circuit and Terry quickly followed in his footsteps, building his own hot rod at the age of 12 (Fig. 2A). He was so driven in this endeavor that he became a founding member of the ‘Vallejo Conquistadores’, a hot rod club in the San Francisco Bay area (Fig. 2B, C). At about the same time, he started to work as a newspaper boy, cleaning walk-in freezers and other low-key jobs; however, like any other teen growing up in California in the 1950s, he enjoyed playing football, water skiing, and rock and rolling with his high school mates. Nonetheless, the overachiever in him also succeeded at school. He liked science and by the 6^th^ grade had become president of the science club (Fig. 3).

As a child, Terry enjoyed comics with stories of superheroes saving the world; later on, he became an avid reader of the stories and adventures of explorers and naturalists. When he discovered the book “The Naturalist on the River Amazons” (Bates 1863), Henry W. Bates’ became Terry’s instant hero. In the book, Bates, a gifted scientist, writer, and illustrator, recounted his adventures in the Amazon, where he spent ten years of his life. Terry dreamed about doing an expedition like that someday.

After finishing high school (1961) in Vallejo Junior College, Terry required some collegiate wanders to find his call to biology, but once found it, he was determined to succeed in his studies. He worked at the Mare Island Shipyard to put himself through college, a dangerous job as in those years, many asbestos-containing products were incorporated into the construction and repair of war submarines and other navy vessels. Asbestos was not known to be toxic until the middle part of the 1970s and he undoubtedly had some exposure. Fortunately for Terry, life took him into a career away from the shipyard. In 1963, Terry married his school sweetheart La Verne Magarian, who was a supportive companion throughout his graduate studies and early career, until their divorce in 1980.

## On the shoulder of giants

**Figure 4.**
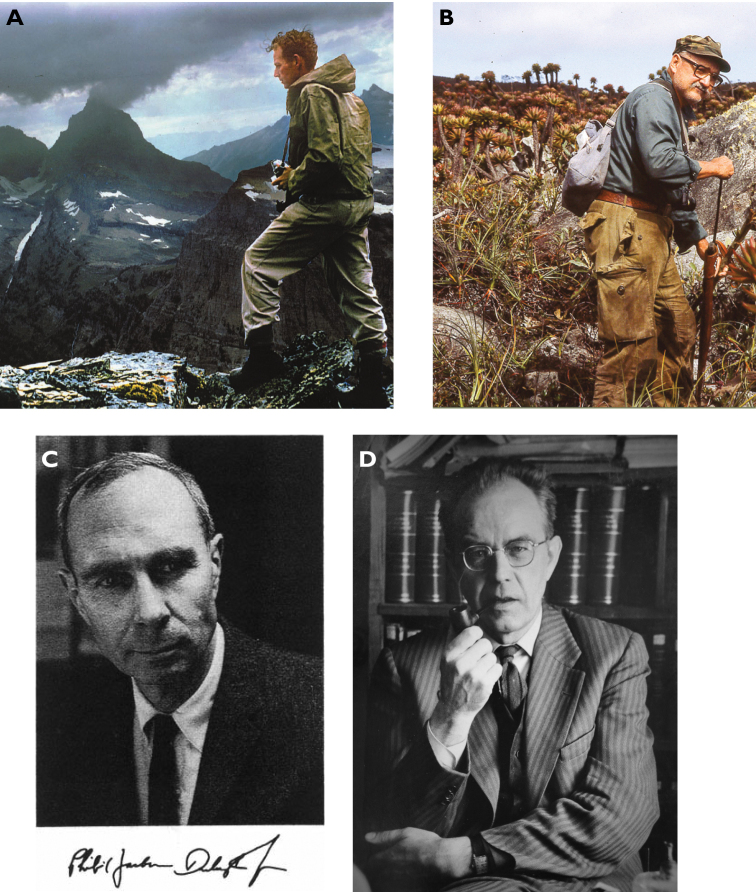
**A** Professor Gordon Edwards (San Jose State University) was the ultimate naturalist and teacher. Terry dedicated *Bembidionedwardsi* to him (photograph: R. Megard) **B** professor George Ball (University of Alberta) in Pico de Neblina Tepui, Venezuela (1983). Terry dedicated many species and the genus *Geballusa* to him (photograph: T. Erwin) **C** Terry did a Post Doc with Professor Philip Darlington at Harvard University (Wilson 1991), and spent a sabbatical at Lund University with **D** professor Carl Lindroth. Darlington, Lindroth, and Ball formed Terry’s “trifecta” in Carabidology (Rice 2017).

Crucial to Terry’s education was the guidance of gifted and insightful professors with whom he shared his curiosity for the natural world and a passion for studying beetles and mentoring. He earned his B.Sc. (1964, Biology) and his M.A. (1966, Biology) degrees from San Jose State College (now San Jose State University) under the guidance of Professor J. Gordon Edwards, whom Terry credited with inspiring his interest in beetles. Edwards, a coleopterist and professional mountain climber, was the ultimate natural history teacher (Fig. 4A). He mentored and influenced more than a hundred students during his teaching career at San Jose (Arnaud et al. 2006). Terry’s master’s thesis was on bombardier beetles from California, a group of carabids that are known for their ability to produce a powerful and hot defensive chemical spray directed at would-be predators. After finishing his M.A., Terry was on the way to study with some of the best carabidologists of his day. For his doctoral studies he went to the University of Alberta, Canada, under the mentorship of Professor George E. Ball (Fig. 4B). Ball was an academic systematist working with carabid beetles and had a profound influence in his graduate students, not only as a scientist and mentor but as the amazing human being he was (Pensoft 2015; Rice 2017; Spence et al. 2019; Kavanaugh 2020).

Terry’s doctoral dissertation, among other publications on bombardier beetles of North and Middle America (Erwin 1967; see list of publications in this volume), opened the door for a Post-Doctoral Fellowship at Harvard University’s Museum of Comparative Zoology with Professor Philip J. Darlington in 1970 (Fig. 4C). Most of Darlington’s research was on the systematics, distribution, and ecology of the carabid beetles, for which he was respected as one of the foremost insect taxonomists in the world. A gifted naturalist, Darlington’s most important contribution to science was his theory of the old-world tropical origin of dominant vertebrate groups which would influence research in zoogeography for a generation (Wilson 1991). In that same year (1970), Terry was hired as Associate Curator in the Department of Entomology at the United States National Museum (now the National Museum of Natural History at the Smithsonian Institution), a position that he accepted on the condition of being able to finish commitments already planned for the year. Terry was granted an “early sabbatical” from the Smithsonian to study with Professor Carl H. Lindroth, at Lund University in Sweden (Fig. 4D). A famous and enormously productive carabidologist, Lindroth was a strong proponent of the glacial refugia hypothesis to explain the distribution of Newfoundland beetles at a time where continental drift was being formulated (Evenden 2021). Lindroth’s studies focused on ground beetles, but had wider biological and geographical implications for science, as he provided ecological evidence to understand the importance of transportation vectors in biological exchanges and the major role played by recurring arrivals of breeding pairs to sustain introductions (a process known today as propagule pressure). He was widely recognized by his popular science lectures, and Sweden television appearances in ‘Fråga Lund’ (“Ask Lund”; Lund referring to the University) where professors and other academics sat in a panel in front of an audience, answering scientific questions. The merit of providing public outreach for his scientific work also influenced Terry considerably.

**Figure 5.**
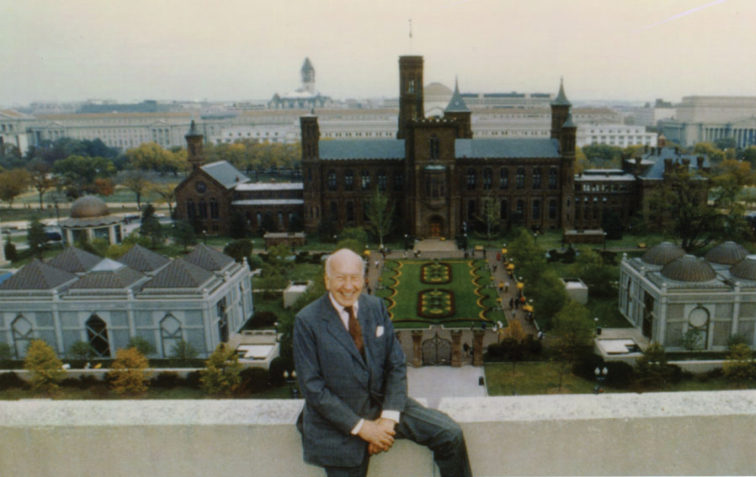
Smithsonian’s Secretary S. Dillon Ripley (1987) (photograph: Smithsonian Institution Archives).

**Figure 6.**
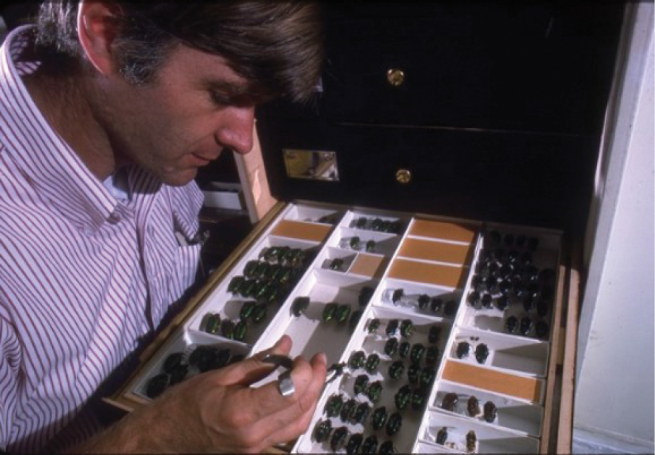
Terry as Assistant Curator at the Entomology Department.

**Figure 7.**
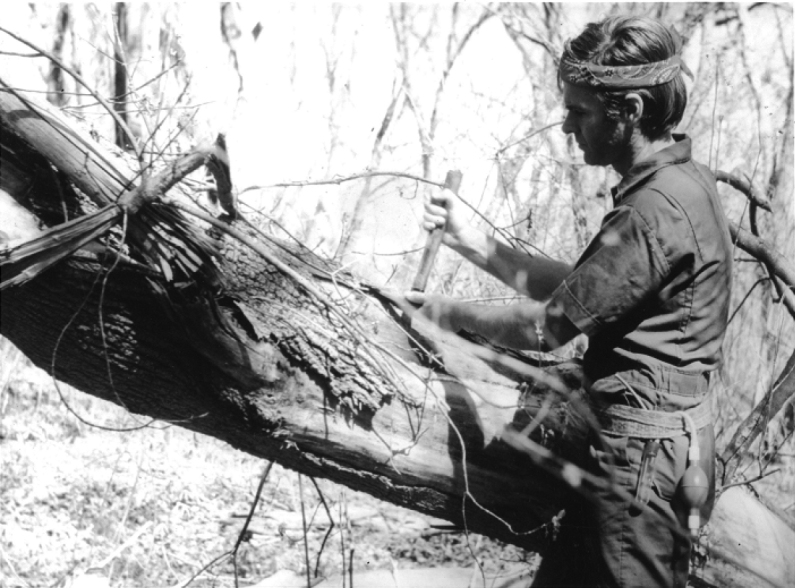
In Barro Colorado Island, Panama, while conducting a systematic study of the ground beetle fauna of Central America (1970) (photographs: Smithsonian Institution Archives).

By the time Terry started his professional life as Associate Curator of Coleoptera, the Smithsonian had become a vibrant educational and cultural institution and was at the peak of its growth due to the vision of Secretary S. Dillon Ripley (Stone 2017) (Fig. 5). From 1964 to 1984, Ripley transformed the institution, adding museums and expanding scientific programs at research centers such as the Smithsonian Tropical Research Institute (STRI) in Panama. The expansion included fellowship programs for permanent staff scientists to encourage and support aspiring tropical biologists. In 1971, after the “early sabbatical” year in Sweden, Terry joined Paul Spangler in the Coleoptera section of the Department of Entomology (Fig. 6). To Terry’s surprise, a research proposal to study Carabidae in California that he had submitted to the Entomology Department before going to Sweden had been approved for him to work at STRI in Panama (Pensoft 2015; Washington Biologists’ Field Club 2020). Thus began a lifetime career on studies of insect biodiversity in neotropical forests (Fig. 7).

By 1975 Terry had conceived a project to explore the forest canopy, one of the richest but least well known and understood environments of the planet, using the fogging technique to collect canopy arthropods (Konop 2015). By fogging arthropods with biodegradable insecticide released into the canopy of *Lueheaseemannii* (Malvaceae) trees in Panama, Terry brought down a rain of thousands of unknown arthropod species that he collected and preserved in alcohol to be sorted later at the lab (Erwin and Scott 1980). The data obtained from these studies would become the basis for his estimate that there were 30 million species of insects worldwide, which brought attention to species diversity and raised public awareness about conservation of tropical forests as centers of megadiversity (Erwin 1982).

In 1976 Terry organized the 1^st^ International Symposium of Carabidology in Washington DC, as part of the activities of the XV International Congress of Entomology (Erwin et al. 1979). More than 100 carabid-lovers attended the symposium including P. Darlington, C. Lindroth, G. Ball, and F. Hieke (East Germany). At the meeting, Darlington gave a talk referring to Newton’s famous statement about doing science standing on the shoulders of giants. This was a message that Terry would carry on throughout his life.

## Terry and the Amazon: the trip was the destination

In 1979, Terry had the opportunity to revive his boyhood dream of visiting the Amazon rainforest, but now as a beetle specialist in the new “Minimum Critical Size of Ecosystems Project (now The Biological Dynamics of Forest Fragments Project) under the lead of Tom Lovejoy. This was a collaborative effort between the Smithsonian Institution and the Instituto Nacional de Pesquisas da Amazônia (INPA, Brazil). The project fostered studies about the consequences of landscape transformation for forest integrity in the central Amazon region as the SLOSS (Single Large or Several Small) debate was at its peak. Researchers attempted to quantify how many species could survive in forest reserves of 1, 10, and 100 ha (Laurance et al. 1997). Terry traveled to Manaus, Brazil, where Terry’s hero, Bates, had been 130 years before. There, Terry used the fogging technique to sample beetles from the canopy of trees (Erwin 1983a, b).

In 1979, Terry also embarked on a new project in the Amazon. Encouraged by his colleague and friend David Pearson (then a professor at Pennsylvania State University), he visited the Explorer’s Inn, one of the first ecolodges on the Tambopata River in southeastern Peru. The site had attracted several tropical biologists such as Al Gentry from the Missouri Botanical Garden and Gerardo Lamas from the Museo de Historia Natural San Marcos (Lima, Peru), both of whom would set world records for the number of plants and butterfly species found in one single locality based on data from the Amazon. Also, in Tambopata Terry met legendary ornithologist Ted Parker from Louisiana State University with whom he shared his love of observations of birds, his other natural history hobby in addition to beetles. Gentry, Parker, Lamas, and Terry, among others later, have established Tambopata Explorer’s Inn as one of the best sites for comparative biodiversity and ecological research in the Amazon. At the time, however, the logistics of doing research in remote locations were cumbersome and chaotic. There was no reliable communication with the outside world from Tambopata, particularly during the rainy season when bad weather caused flight cancellations and resulting shortages of supplies that could limit research for days or even weeks at a time. Working there was a difficult task on a limited research budget. In 1985, after a devastating fire in the central building of the lodge, a season’s worth of Terry’s valuable insect collection, field notes, and equipment burned despite the efforts of everybody present at the station.

Terry, Gentry, and Parker would later reunite in St. Louis, Missouri in 1992, when Terry took a sabbatical year while I was doing my M.S. at the University of Missouri at St. Louis. Every Friday afternoon, Gentry held an open botany class at the Missouri Botanical Garden that everyone attended, including Terry and Ted. After the session, the “beer hour” was announced by loudspeaker in a recorded message translated to every language in the world and many informal but highly stimulating science discussions ensued. Terry and I also much enjoyed the opportunity to observe birds with Ted on the weekends. Unfortunately, a plane accident took the lives of Gentry and Parker in 1993 while they were searching for a new locality for the Rapid Assessment Program from Conservation International. The sense of loss hit us hard, but overall, the botanical, ornithological, and conservation communities were devastated.

Terry dramatically expanded our concept of terrestrial insect diversity with the publication of “Tropical forests: their richness in Coleoptera and other arthropod species” (Erwin 1982). In this paper, based on data from his fogging work, Terry hypothesized that there were as many as 30 million arthropod species worldwide, an order of magnitude more than existing estimates that predicted approximately one million species. Based on the idea that there were so many unknown species, the conservation community embraced the message that the world was likely losing many more species than previously imagined. Since then, the fogging technique has been used by many to gather data for additional estimates the number of species on the planet. Terry’s last estimate of the number of insect species was ‘gazillions’ (García-Robledo 2020).

During the 1980’s the study of biological diversity had steadily picked up momentum and publications regarding species diversity, deforestation rates, species extinction, and fragmentation were abundant. Furthermore, both the scientific community and the general public began to appreciate the close linkage between conservation and economic development. In this context, in 1986, Walter G. Rosen, from the National Academy of Sciences, and Edward W. Bastian, from the Smithsonian Institution, organized ‘The National Forum on Biological Diversity’ (Washington, D.C., 21–24 September). Terry and 70 other leading participants converged at this session, bringing huge scientific expertise, interdisciplinary commitment, and conservation concern to the forum (Erwin 1988). A contracted form of the phrase ‘biological diversity’ was used by Dr. Rosen during the forum, and this gave the name “Biodiversity” to the book of proceedings published by the National Academy Press two years later (Wilson and Peter 1988) and a term that bracketed a whole new field of scientific endeavor that had become a major investment of Terry’s scientific career.

**Figure 8.**
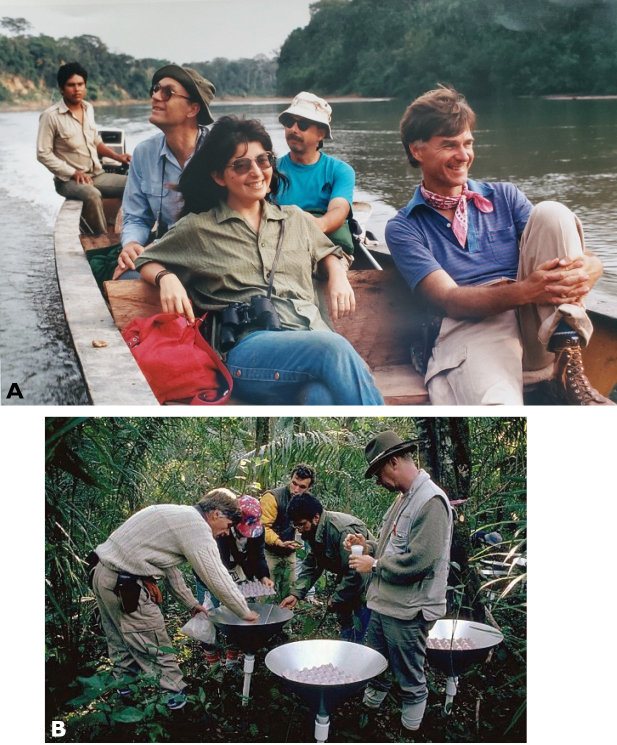
**A** Setting insect traps before fogging (BIOLAT Program. Photos: Chip Clark, 1987) **B** on the Manu river with David Pearson (Arizona State University), Wayne Mathis (NMNH, Smithsonian Institution), and Grace Servat (Museo de Historia Natural, Universidad San Marcos).

**Figure 9.**
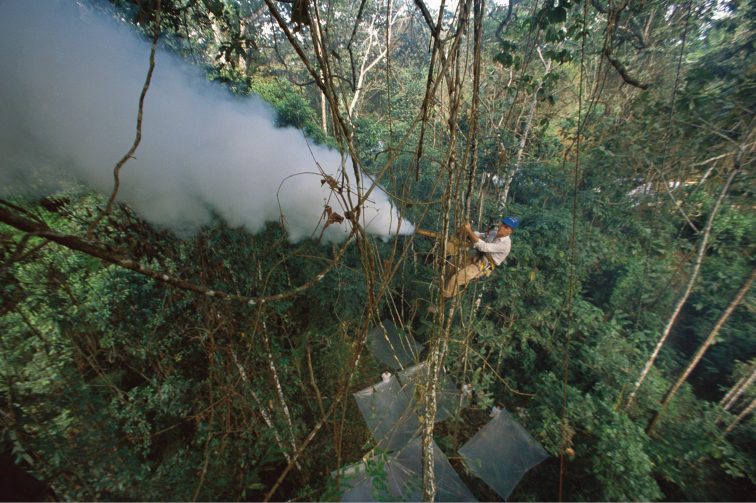
Canopy fogging at B. Pacaya Samiria, Peru (photograph: Mark Moffett).

During 1986, Terry was appointed Director of the Biological Diversity in Latin America Program (BIOLAT), a new initiative of the Smithsonian Institution and the United Nations Educational, Scientific and Cultural Organization (UNESCO) (Miller in D’Souza 2020). The project’s aim was to document the flora and fauna of high-diversity sites in tropical forests on a long-term basis using standardized sampling. In 1987 the BIOLAT Program was launched at Beni National Park (Bolivia) and Pakitza in Manu Biosphere Reserve (Peru) (8A). I met Terry as part of the BIOLAT team at Pakitza where I was a student associated with the Bird Department at the Natural History Museum of San Marcos University in Lima, Peru (Fig. 8B). Terry was the director of BIOLAT until 1989, and remained doing research in Pakitza until 1993. At the same time, we organized other expeditions to little-explored places such as the Pacaya Samiria National Reserve (Fig. 9) and the Sucusari River in Loreto, Peru, when major economic, political, and social unrest reached a peak in Peru.

## Students and tigers

In 1994, Terry did some environmental impact studies at the Onkone Gare Station in Yasuní National Park, Ecuador, one of the last high-biodiversity protected areas in the western Amazon (Bass et al. 2010). Along with other well-recognized scientists, he participated in a study that documented the impacts on the flora and fauna caused by the construction of a 150-kilometer road for oil transportation by the Maxus oil consortium (Swing, this volume). One of the outcomes of the study was the estimate that a single hectare of forest in Yasuní may contain 100,000 insect species. During the years that followed and to Terry’s great sorrow, Yasuní become emblematic of the environmental threats facing the entire Amazon region.

**Figure 10.**
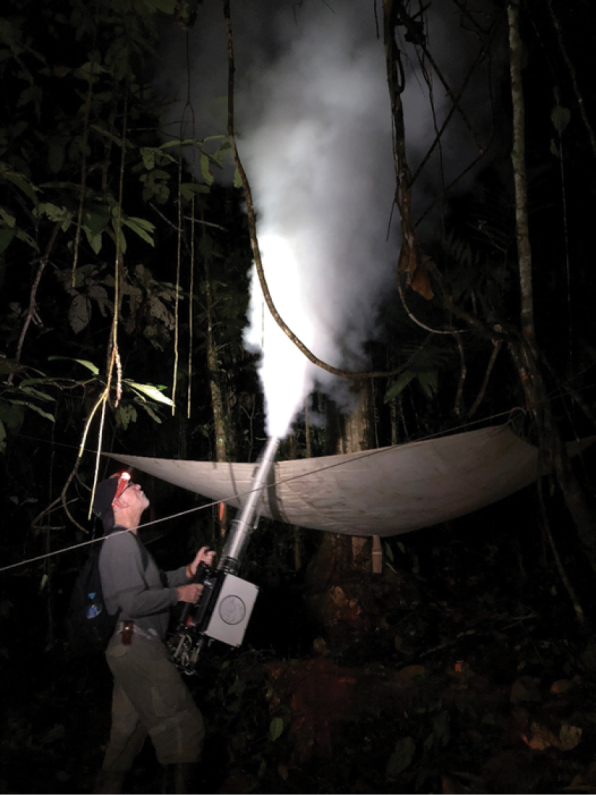
Tiputini Biological Station (photograph: B. Garner).

Terry’s long-term program to monitor insect diversity at Tiputini’s Biological Station (TBS; Fig. 10), a locality adjacent to Yasuní National Park, attracted a large number of young and enthusiastic biologists, some of them, among them, to which he introduced to the wonders of tropical forests (Boyd; Maveety; Riley; this volume). The TBS is a 1,500-acre center for education, scientific research, and conservation, developed by Boston University and the Universidad San Francisco de Quito. Terry had a special placed in his heart for Tiputini and the work environment there involving students, faculty, researchers, and ‘the tigers’ (as the personnel at the station called themselves) (Mosquera; Swing; this volume). His last trip to Yasuní was in June 2019.

Terry’s commitment to students was a priority in his life, he never missed an opportunity to participate as invited professor in field courses for the Organization for Tropical Studies (OTS) in Costa Rica and Peru; or to serve as external advisor in graduate student committees (Riley; Maveety; Zamorano; this volume). Through his students he developed associations with many universities in the US and overseas. Terry was always eager to provide opportunities for young scientists, hosting interns and fellows at the Department of Entomology at the Smithsonian, sponsoring them to attend meetings and conferences, and trying to connect them further according to their interests (Simms; Garner; this volume). He really believed he stood on the shoulders of giants and kept reaching down and pulling up younger scientists to stand on his shoulders (Neisbitt in D’Souza 2020).

## Other key points

- 1971–2020. Terry work as curator of Coleoptera at the National Museum of Natural History for more than 50 years. By the time he passed he was the only curator serving the Smithsonian Institution in charge of one of the largest beetle collections in the world, numbering ca. 12 million specimens. Terry alone contributed with more than five million specimens collected from the canopy of Neotropical rain-forests (https://www.smithsonianofi.com/department-of-entomology/).
- 1972. Terry was elected to The Washington Biologists’ Field Club, a nonprofit organization created in 1901 to study and promote the study of biology in the Washington, DC area. The Club’s field headquarters are in Plummer’s Island, Maryland (Steiner, this volume). Terry served as Secretary from 1986–1987 and on the Research Committee (Washington’s Biologists Field Club 2020). He published a natural history of the carabid beetles of Plummer’s Island (Erwin 1981) with an analysis of the fauna using specimens that had been collected by many of the founding members, and which had been stored unstudied in the Smithsonian’s collections.
- 2000–2002. Terry was a Government Board Member and Scientific advisor to the All-Species Foundation an organization aimed to catalog all species on earth. The All-Species Foundation was regarded as an important step forward in expanding, modernizing, and digitizing the field of taxonomy (Gewin 2002). Among the many initiatives born from the All-Species meetings was to provide a descriptive webpage for every species. This initiative materialized in 2008 in the Encyclopedia of Life (EOL). EOL gathers information from resources across the world such as museums, learned societies, expert scientists, and others into one massive database and a single, easy-to-use online portal (http://eol.org). EOL was launched by the Field Museum, Harvard University, the Marine Biological Laboratory, the Missouri Botanical Garden, and the Smithsonian Institution. Terry was among the first to contribute to EOL with data ca. 10,000 species of carabid beetles from his series of carabid books for barcoding. Carabidae was one of the first families covered in the project and deposited on the Barcode of Life Data Systems, thus making specimens discoverable and accessible to the world’s scientific community (García-Robledo et al. 2013, 2020; Miller in D’Souza 2020).
- 2008–2020. Terry served as Editor in Chief of ZooKeys, an innovative open access journal in taxonomy and systematics that has accelerated biodiversity research using new tools for descriptive taxonomy (Penev et al. 2008, 2009a, b, 2010a, b, 2011, 2012a, b; Stoev et al. 2011, 2013; Erwin et al. 2015, 2018). The ZooKeys framework developed by the Pensoft team lead by Lyubomir Penev had Terry’s full support from the beginning and was the source for fruitful initiatives, great scientific discussions, and wonderful memories (Penev, this volume).
- One of the last innovative research projects in which Terry was willing to participate as co-sponsor (with John Kress in the Botany Department) was the Post-Doctoral research of Carlos García-Robledo. García-Robledo’s project documented plant species (Zingiberales) eaten by 20 species of beetles (previously selected and identified). He had created a baseline library of DNA barcodes for each beetle and the plant species eaten in the insect herbivore-plant network. The DNA barcodes were then used to identify the diets of beetle species at locations along an altitudinal gradient to model the ecology and evolution of plant-herbivore interactions under the effects of climate change on plant extinctions and the co-extinctions of associated insect herbivores (García-Robledo et al. 2013). For García-Robledo and the members of his research team Terry was considered a very Agra dable collaborator, mentor, and friend (‘agradable’ means nice in Spanish) (García Robledo 2020; García-Robledo et al. 2020).

## Popularization of science

**Figure 11.**
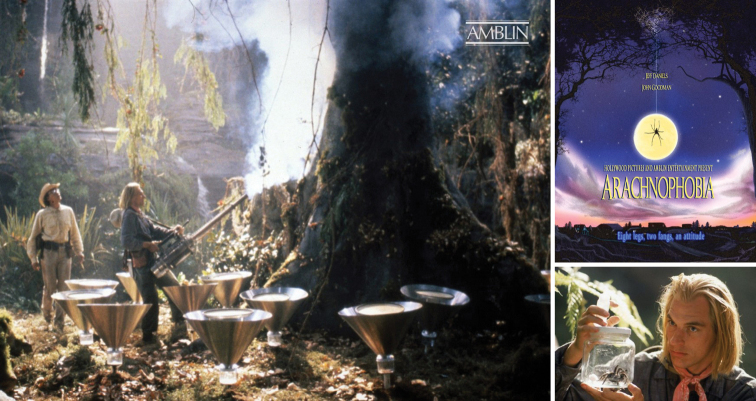
Terry’s fogging as depicted in the movie Arachnophobia… down to the pink bandana! (photographs: https://amblin.com/movie/arachnophobia/Amblin).

Terry’s fogging technique for collection of canopy arthropods was pretty spectacular. He appeared in National Geographic and the Discovery Channel documentaries, that he later recommended to film director, Steven Spielberg, when asked for advice about the first sequence of the movie “Arachnophobia”. The movie not only recreated very well the fogging of trees in a tropical forest, but they actually replicated the clothes Terry was wearing … right up to the pink bandana around the neck! (Fig. 11).

**Figure 12.**
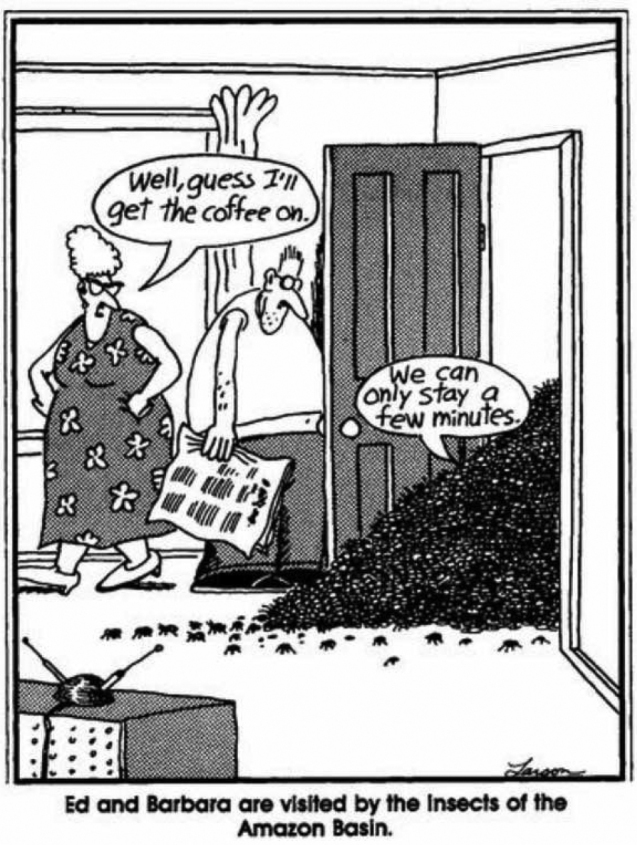
Larson’s Far Side in regard of Terry’s estimate of 30 million insect species (1985).

**Figure 13.**
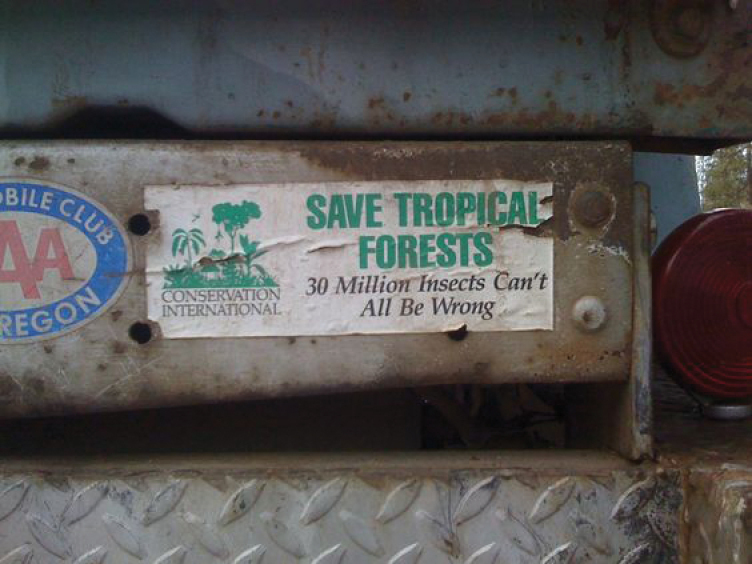
Bumper sticker from Conservation International.

Terry’s publication about the “30 million species” reached the public in unexpected ways. Gary Larson a popular cartoonist, produced a couple of single-panel cartoons published in newspapers in the 1980s that built on this theme (Fig. 12). Also, in the 1990s, Conservation International made the popular bumper sticker: “Save tropical forests, 30 million insects can’t all be wrong” (Fig. 13).

**Figure 14.**
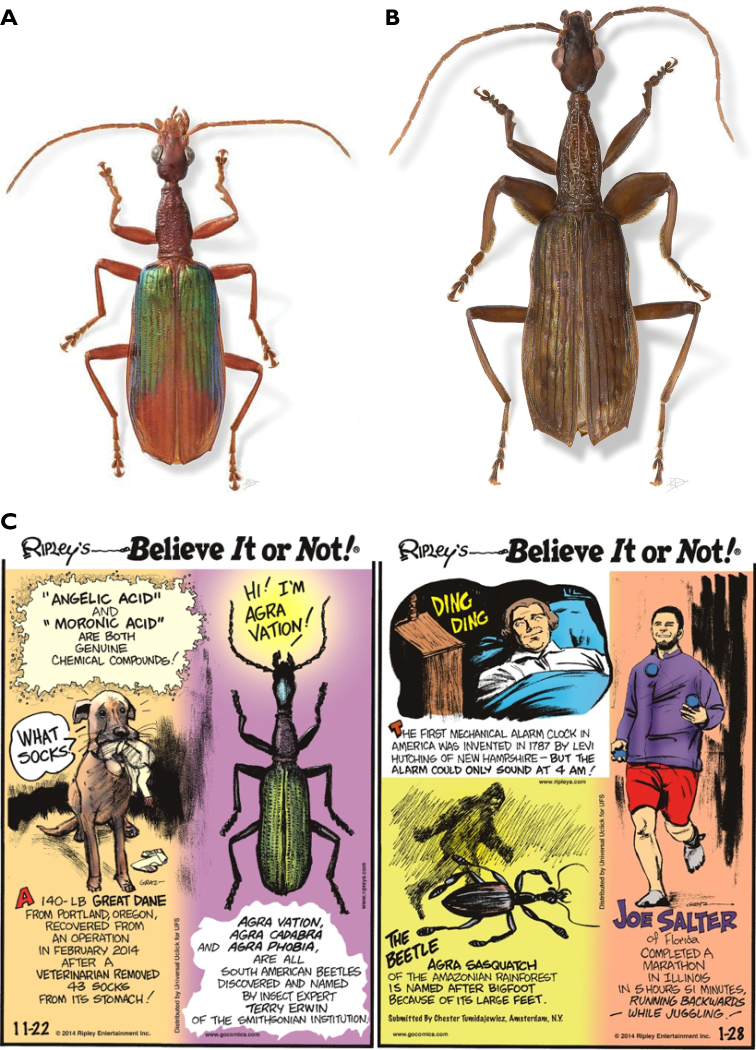
**A***Agrakatewinsletae* Erwin, 2002 dedicated to Kate Winslet from ‘Titanic’. “Her character did not go down with the ship, but we will not be able to say the same for this elegant canopy species, if all the rain forest is converted to pastures” **B***Agraschwarzeneggeri* Erwin, 2002 dedicated to Arnold Schwarzenegger, in reference to the “markedly developed (biceps-like) middle femora of the males of this species reminiscent of the actor’s physique” **C** scientific names in ‘Ripley’s Believe it or Not’.

As characterizes any good taxonomist, Terry considered that species were not just names, points on an evolutionary tree, nor abstract sequences of DNA, but rather encode the results of countless millennia of complex interactions and processes. To understand the diversity of life on Earth, and the true rate at which species are disappearing, Terry started by naming each new carabid falling from the canopy. Describing and naming species is the first step to unlock, through additional scientific work, the details about their biology, evolutionary relationships with other species, and a functional understanding of the complex tropical ecosystem. Naming species is a serious business in its own right, but it does not mean taxonomists cannot have some fun doing it, particularly if they are sharp and witty. Terry collected so many new species that after he arranged them in groups for revision, he could dedicate them through their names to scientists (e.g., the genus *Batesiana* to Bates, *Agraeowilsonii* to E. O. Wilson, etc.); professors (e.g., *Bembidionedwardsi* to J. G. Edwards, the genus *Geballusa* to G. E. Ball, etc.); friends (e.g., *Brachinuskavanaughi* to D. Kavanaugh, *Valeriaaschero* to Valeria Aschero), family (e.g., *Agragrace* to me), and collecting localities (e.g., *Asklepiapakitza*, to the Pakitza station in Manu National Park, *Asklepiabiolat* to the BIOLAT project, the genus *Inpa* to the Instituto Nacional de Pesquisas da Amazonia, *Hybopteratiputini* to Tiputini Biological Station, etc.). He also accepted suggestions from colleagues and used plays on words (e.g., *Agracadabra*, *Agravation*, etc.) (Steiner, this volume). In his search for new and unique names, he started dedicating species to stars in movies such as Titanic (*Agrakatewinsletae* to Kate Winslet), Armageddon (*Agraliv* to Liv Tyler), or just to emphasize a physical attribute (*Agraschwarzeneggeri* to Arnold Schwarzenegger in recognition of the swollen biceps-like middle femora) (Fig. 14A, B). Terry’s names grabbed public attention and some were included in ‘Ripley’s Believe it or Not’ (Fig. 14C).

Terry’s endless motivation and energy was borne of a sense of wonder, enjoyment, satisfaction, and challenge from his everyday work on the beetle collections. He was very proud to introduce himself as a ‘taxonomist’; however, his contribution to the world of science and conservation of tropical forests went much further than simply naming and counting species. Terry was a pioneer in neotropical conservation biology and canopy research. His holistic approach to field biology, with Carabidae at its core, enabled him to understand the relatedness of species as well as the mechanisms that drive the evolution of such incredible diversity. Terry’s thoughts will live on in the many scientists, educators, environmental advocates, and nature lovers that he influenced both through direct contact and his scientific work.
